# Bony abnormalities of the hip joint: a new comprehensive, reliable and radiation-free measurement method using magnetic resonance imaging

**DOI:** 10.1093/jhps/hnu009

**Published:** 2014-10-07

**Authors:** Marcie Harris-Hayes, Paul K. Commean, Jacqueline D. Patterson, John C. Clohisy, Travis J. Hillen

**Affiliations:** ^1^Program in Physical Therapy and Department of Orthopaedic Surgery, Washington University School of Medicine, 4444 Forest Park, Campus Box 8502, St. Louis, MO 63108, USA, ^2^Electronic Radiology Laboratory, Mallinckrodt Institute of Radiology, Washington University School of Medicine, Campus Box 8131, St Louis, MO 63110, USA, ^3^Department of Orthopaedic Surgery, Washington University School of Medicine, 660 S. Euclid, Suite 11300 West Pavilion, Campus Box 8233, St Louis, MO 63110, USA and ^4^Department of Radiology, Washington University School of Medicine, 660 S. Euclid, Campus Box 8131, St Louis, MO 63110, USA

## Abstract

The objective of this study was to develop comprehensive and reliable radiation-free methods to quantify femoral and acetabular morphology using magnetic resonance imaging (MRI).

Thirty-two hips [16 subjects, 6 with intra-articular hip disorder (IAHD); 10 controls] were included. A 1.5-T magnetic resonance system was used to obtain three-dimensional fat-suppressed gradient-echo images at the pelvis and distal femora. After acquisition, pelvic images were post-processed to correct for coronal, axial and sagittal rotation. Measurements performed included acetabular version (AV), femoral version (FV), lateral center-edge angle (LCEA), femoral neck angle (FNA) and alpha angle (AA) at 3, 2, 1 and 12 a.m. Two experienced raters, a musculoskeletal radiologist and an orthopedic physical therapist, and a novice rater, a research assistant, completed reliability testing. Raters measured all hips twice with minimum 2 weeks between sessions. Intra-class Correlation Coefficients (ICCs) were used to determine rater reliability; standard error of measurements was reported to estimate the reasonable limits of the expected error in the different raters’ scores.

Inter-rater reliability was good to excellent for all raters for AV, FV, FNA and LCEA (ICCs: 0.82–0.98); good to excellent between experienced raters (ICCs: 0.78–0.86) and poor to good between novice and experienced raters (ICCs: 0.23–0.78) for AA. Intra-rater reliability was good to excellent for all raters for AV, FV and FNA (ICCs: 0.93–0.99); for one experienced and novice rater for LCEA (ICCs: 0.84–0.89); moderate to excellent for the experienced raters for AA (ICCs: 0.72-0.89). Intra-rater reliability was poor for the second experienced rater for LCEA (ICC: 0.56), due to a single measurement error and for the novice rater for AA (ICCs: 0.17–0.38).

We described MRI methods to comprehensively assess femoral and acetabular morphology. Measurements such as AV, FV and FNA and the LCEA can be made reliably by both experienced and novice raters; however, the AA measurement was reliable only among experienced raters.

## INTRODUCTION

Intra-articular hip disorders (IAHD) are a major cause of hip joint pain and possible precursor to hip osteoarthritis [1–4]. Recent emphasis has been placed on femoral and acetabular abnormalities, such as a reduced head–neck offset and shallow acetabulum, as contributors of IAHD [5, 6], yet the relationship between bony abnormalities and joint pathology is still under investigation. To better understand the relationship between femoral and acetabular morphology and IAHD, safe and reliable methods to assess bony morphology in symptomatic and asymptomatic people are needed. Additionally, when hip preservation surgery is considered, three-dimensional (3D) imaging can assist in determining the procedure type and details of the surgical correction.

Given the hip joint’s proximity to reproductive organs and risk of exposing these organs to radiation, a method that uses no ionizing radiation would be preferred, particularly when performing investigations in an asymptomatic population. Magnetic resonance imaging (MRI) provides a safe, radiation-free method to assess bony morphology. 3D sequences can be collected in a relatively short time period. The acquired images can then be post-processed in various imaging planes and measurements performed on those post-processed images. Multiple measurements can be made, including measures of femoral head–neck junction morphology and acetabular coverage of the femoral head, thus allowing a comprehensive study of femoral and pelvic morphology.

When assessing acetabular coverage of the femoral head, it is important to standardize pelvic position [7–12]. Modeling studies have reported significant inflation in values of acetabular version (AV), when excessive pelvic obliquity or tilting is present [10, 11]. Studies using computed tomography (CT) and radiographs suggest that subject malpositioning directly affects measures of acetabular coverage of the femoral head [11–14]. Despite this clear need for standard pelvic orientation, obtaining optimal subject alignment during image acquisition can be challenging. Specific guidelines have been developed to assess pelvic orientation prior to performing measurements of acetabular coverage using radiographs [15]. Methods to correct pelvic orientation using CT have been introduced [16], but are not widely used. The 3D nature of MRI allows for standardization of pelvic orientation using specific bony landmarks after images have been acquired; however, methods to standardize pelvic orientation have not been described in previous studies using MRI.

The purpose of this study was to develop a comprehensive, reliable and radiation-free measurement method using MRI to quantify the femoral and acetabular morphology. We used MRI to acquire 3D images providing the ability to view various planes and obtain multiple bony measurements including AV, femoral version (FV), femoral neck angle (FNA), acetabular lateral center-edge angle (LCEA) and alpha angle (AA) representing the head–neck junction at various femoral neck positions. To reduce the effect of subject positioning on acetabular variables, we standardized pelvic orientation prior to performing the acetabular measurements. Additionally, we described the training associated with performing the bony morphology measurements and assessed the reliability of experienced and novice raters. We hypothesized that with specific training, the rater reliability between experienced and novice raters would be high.

## MATERIALS AND METHODS

This study was approved by the Human Research Protection Office of Washington University School of Medicine. All subjects read and signed an informed consent statement before participating in the study. The Guidelines for Reporting Reliability and Agreement Studies were used for reporting [17].

### Subject selection and clinical data

The subjects in this study were the first 10 control subjects and first 6 subjects with IAHD enrolled (January 2011 to March 2012) in a prospective cohort study developed to assess musculoskeletal differences between people with and without IAHD. Subjects with and without IAHD, 18- to 60-years old, were recruited from the Washington University School of Medicine's Orthopedic, Physical Medicine and Rehabilitation, and Physical Therapy clinics and Washington University School of Medicine's research volunteer database and through public announcements. People with IAHD reported deep joint or anterior groin pain that was reproducible with the Flexion– Adduction– Internal Rotation impingement test, also known as the FAIR or FADIR test [18]. People without IAHD reported no history of hip pain. Exclusion criteria for both groups included the following: (i) previous hip surgery or fracture, (ii) any contraindication to MRI, (iii) known pregnancy, (iv) neurological involvement that influenced balance and (v) body mass index (BMI) greater than 30. Two exclusion criteria, neurological involvement and BMI, were necessary for other testing procedures used in the parent study. After consent was obtained, Rater 2 (MHH) completed subjective history and performed a clinical examination to confirm the presence of IAHD in subjects with IAHD. For the purposes of this study, subjects were enrolled into the IAHD group based on patient’s report of symptoms and physical examination. Subjects were excluded if screening tests for differential diagnosis was positive indicating possible lumbar spine radiculopathy. We did not exclude patients if they reported additional symptoms consistent with extra-articular sources. The subject was then escorted to the imaging center for the MRI.

### Image acquisition and post-processing

A 1.5-T magnetic resonance system (Avanto; Siemens, Erlangen, Germany) was used to obtain two 3D fat-suppressed gradient-echo imaging sequences, one centered at the pelvis and one centered at the femoral condyles. For image acquisition, each subject was supine on the MRI table with the lower extremities in neutral (0° hip flexion, 0° hip abduction, and 0° hip rotation). Prior to placement of the coils, standardized methods were used to optimize subject positioning. From the hooklying position, the subject was asked to perform a bridging technique and return to supine with legs extended. A brief, traction maneuver was applied by Rater 2, by grasping the subject’s ankles and pulling inferiorly on both lower extremities. Visual appraisal and palpation were used to assess the subject’s position. A peripheral angiography coil overlying the lower extremities, a body matrix coil overlying the pelvis and the spine coil were used during imaging. Straps were used to secure the coils and minimize subject movement. Spacers were also placed around the feet to maintain the neutral position of the lower extremities. Scout images of the pelvis and distal femurs were obtained to identify the capture volume. 3D fat-suppressed gradient-echo sequences Double echo steady state (DESS) were acquired in the coronal plane at the pelvis and the distal femora. The following parameters were used: slice thickness 0.82 mm, Repetition time (TR) 15.96 ms, Echo time (TE) 6.2 ms, Field of view (FOV) 400 mm at the pelvis and distal femora, 512 × 512 matrix and total imaging time for both sequences was approximately 14 min.

**Fig. 1.**
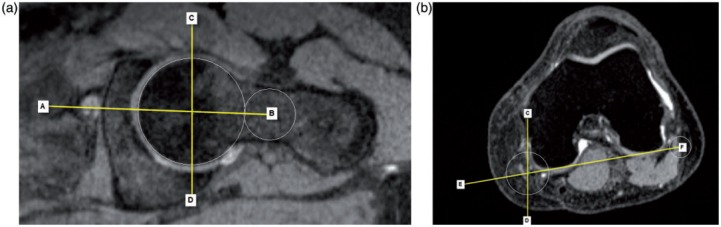
FV is the relative rotation between the femoral neck and femoral shaft. FV is represented as the angle between (a) line AB that extends through the femoral head center and bisects the proximal femoral neck and (b) line EF that aligns with the distal femoral condyles. A vertical reference line CD perpendicular to the coronal axis of the pelvis is used to assist with angle calculation.

**Fig. 2.**
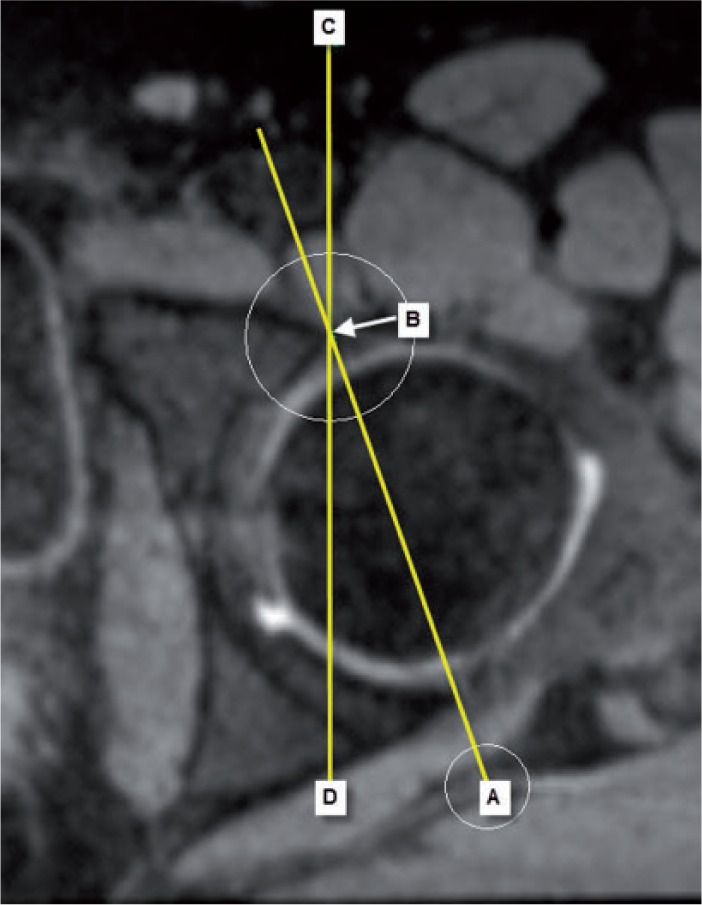
AV describes the extent the acetabulum surrounds the femoral head in the transverse plane. The AV angle defined by points ABD is formed by line AB connecting the anterior and posterior acetabular rims and vertical line CD perpendicular to the coronal axis of the pelvis.

**Fig. 3.**
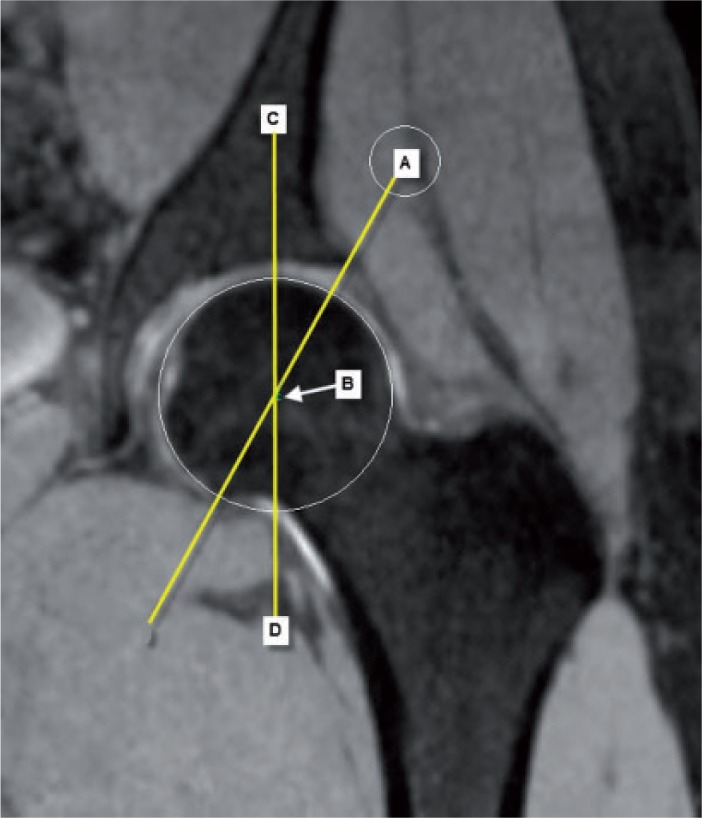
LCEA represents the superolateral femoral head coverage provided by the acetabulum. The LCEA is defined by points ABC and is formed by line CD perpendicular to the transverse axis of the pelvis drawn from femoral head center and line BA line from the femoral head center to the superolateral point of acetabulum.

**Fig. 4.**
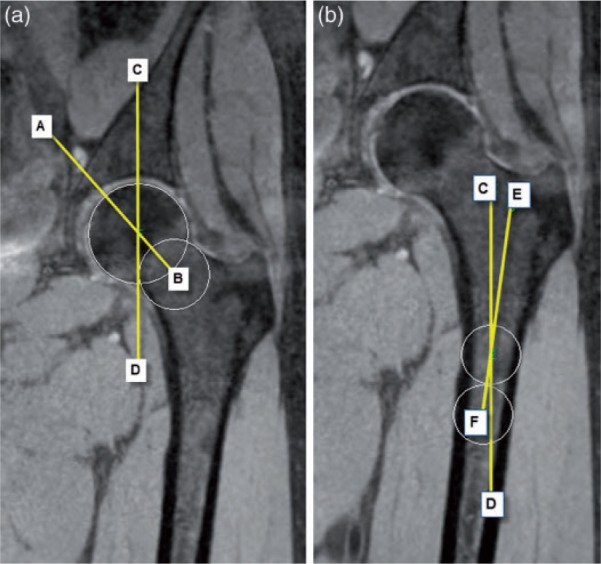
FNA is formed by (a) line AB that extends through the femoral head center and bisects the proximal femoral neck and (b) line EF bisecting the femoral shaft. A vertical reference line CD perpendicular to the transverse axis of the pelvis is used to assist with angle calculation.

**Fig. 5.**
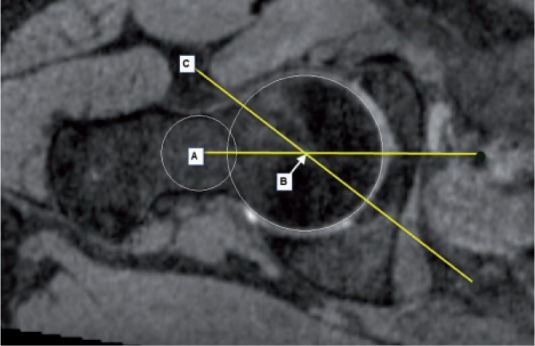
AA represents the femoral head–neck junction concavity. The 3 a.m. position is shown. The AA is defined by points ABC and is formed by line BC from the femoral head center to the point on the anterolateral head–neck junction where the radius of the femoral neck first becomes greater than the radius of the femoral head and line BA line drawn from the femoral head center through the center of the femoral neck.

Using an independent workstation (LEONARDO; Siemens), the 3D MR images were post-processed to create two-dimensional (2D) pelvic images used for making the study measurements (Figs 1–5). The proximal image for FV was selected first and saved as a single 2D image (Fig. 1a). To standardize the pelvic orientation across all subjects, the 3D pelvic images were post-processed via 3D image manipulation to correct for pelvic rotations in the following order: coronal, axial and sagittal. Correction for rotation in the coronal plane was made by aligning the inferior margins of the ischial tuberosities. Next, correction for rotation in the axial plane was made by aligning the bilateral posterior acetabular walls. Finally, correction for rotation in the sagittal plane was made by aligning the anterior superior iliac spine and ipsilateral anterior pubic symphysis. After the 3D image manipulation, a single 2D image was created and saved for each of the following measurements: AV (Fig. 2), LCEA (Fig. 3) and FNA (Fig. 4). Finally, a radial reformat was performed along the femoral neck axis at 30° intervals [19] to obtain images for the AA measurement at 12, 1, 2 and 3 o'clock; 12 o'clock indicates the superior (lateral) location and 3 o'clock indicates the anterior location (Fig. 5). Total time for post-processing and 2D image selection was approximately 15 min per subject.

Once post-processing was complete, the images were saved into a secure server. Images were then downloaded from the server to a desktop computer. A research assistant not involved in the reliability testing renamed and saved each image into a new file to blind the raters to the original subject number and therefore the subject group, IAHD or asymptomatic control.

### Rater training and measurement

Two experienced raters and one novice rater participated in reliability testing. Rater 1 (TJH) is a board-certified radiologist with 4 years of clinical experience in musculoskeletal radiology. Rater 2 (MHH) is a board-certified clinical specialist in orthopedic physical therapy with 15 years of clinical experience and 4 years of research experience with specific focus on the hip. Rater 3 (JDP) is a research assistant with 1 year of graduate training.

Raters 1 and 2 along with a research engineer (PKC) and an MR technologist were involved in developing the MR sequence, post-processing and measurement techniques. Methods for measurement are presented in Figs 1–5. A written manual describing the measurement methods was developed and used by all raters during reliability testing. After initial development of the manual, the experienced raters independently completed five practice sessions, and then compared their measurements. The written manual was revised and finalized based on these comparisons. Training for Rater 3 included self-study of the written manual, followed by a demonstration of the methods by Rater 2. Rater 3 then participated in a monitored practice session with immediate feedback, followed by five independent practice sessions. Feedback and discussion were provided after each practice session. None of the images used during the practice sessions were used in the reliability testing.

### Rater reliability

To assess rater reliability, the three raters independently completed repeat measurements using the described procedures. Images were imported into ANALYZE 11.0 software [20] (Biomedical Imaging Resource, Mayo Foundation, Rochester, MN) for measurement. Each variable was measured only once per rater, per session. To reduce rater recall, the second session of measures was performed at least 2 weeks after the first measurement. Discussion among the raters was not allowed once the reliability assessment began. Each rater was blinded to the subject’s group (IAHD or control); however, all raters were aware their measurements were being used to assess reliability. The first session of each rater was used to assess inter-rater reliability.

### Statistical analysis

Statistical analysis was performed using SPSS Statistics 20.0 for Windows (SPSS Inc., Chicago, IL, USA). Intra-class Correlation Coefficients (ICCs 2, 1) were used to determine the intra-rater and inter-rater reliability. The ICCs provide an index that reflects both the degree of correspondence and agreement among ratings [21]. Standard error of the measurement (SEM) was used to estimate the reasonable limits of expected error in the different raters’ scores, therefore it is useful in the interpretation of the reliability of a measurement.

## RESULTS

### Subjects

Sixteen subjects, 6 with IAHD and 10 controls, were enrolled. Complete demographic information is provided in Table I. One hip of one subject with IAHD was excluded from AA measures due to marrow edema at the femoral head–neck junction and adjacent soft tissues resulting in difficulty visualizing the cortical margin used to make the measurement.

**Table I.** Subject characteristics

Variable	Control	IAHD
	(*n* = 10)	(*n* = 6)
Sex	2M:8F	6F
Age (years)^a^	30.5 ± 12.3	32.6 ± 11.7
BMI (kg/m^2^)^a^	24.9 ± 3.0	24.0 ± 3.5

F = female; M = male.

^a^Values are mean ± standard deviation.

### Intra-rater reliability

Intra-rater reliability, Table II, was good to excellent for all raters for AV, FV and FNA and for Rater 1 and Rater 3 for LCEA. Rater 2, however, demonstrated poor reliability for LCEA, due to a single measurement error. Intra-rater reliability for AA ranged from moderate to excellent for Rater 1 and Rater 2 and was poor for Rater 3.

**Table II.** Intra-rater reliability of all testers

Variable	*n*^a^	Intra-tester					
		Tester 1		Tester 2		Tester 3	
		ICC (2, 1)	95% CI	ICC (2, 1)	95% CI	ICC (2, 1)	95% CI
Acetabular version	32	0.96	0.92–0.98	0.96	0.82–0.98	0.94	0.87–0.97
Femoral version	32	0.99	0.97–0.99	0.96	0.91–0.98	0.99	0.98–0.99
Femoral neck angle	32	0.97	0.94–0.99	0.97	0.94–0.99	0.93	0.85–0.96
Lateral center-edge angle	32	0.89	0.79–0.95	0.56^b^	0.25–0.76	0.84	0.70–0.92
Alpha angle 3	31	0.87	0.75–0.94	0.84	0.70–0.92	0.22	−0.10 to 0.52
Alpha angle 2	31	0.78	0.58–0.89	0.75	0.52–0.87	0.38	−0.03 to 0.65
Alpha angle 1	31	0.89	0.78–0.94	0.83	0.66–0.92	0.28	−0.10 to 0.60
Alpha angle 12	31	0.72	0.49–0.86	0.84	0.48–0.92	0.17	−0.10 to 0.46

CI = confidence interval.

^a^For alpha angle, tester 1 and tester 2 independently determined the scans from one hip was insufficient to measure due to bone marrow and soft tissue edema at the femoral head–neck junction, and therefore this scan was excluded from the analysis. ^b^Upon review of images after testing, it was noted that tester 2 made a significant error on the second measurement of the LCEA. With removal of this error, ICCS:LCEA = 0.82 (0.61–0.91).

### Inter-rater reliability

Inter-rater reliability and SEMs are provided in Tables III and IV. Inter-rater reliability was good to excellent for all raters for AV, FV, FNA and LCEA. Additionally, inter-rater reliability was good to excellent between the experienced raters (Raters 1 and 2) for AA. Between the experienced raters and the novice rater (Rater 3), reliability was poor for the AA measurements. The SEMs for AV, FV, FNA and LCEA ranged from 1.1° to 2.0° and AA from 2.2° to 3.1°.

**Table III.** Inter-rater reliability, SEM, means and standard deviations for measurements completed by experienced testers

Variable	*n*^a^	Inter-tester					
		Tester 1, Tester 2					
		ICC (2, 1)	95% CI	SEM (°)	95% SEM (°)	Mean (°)	SD (°)
Acetabular version	32	0.94	0.79–0.98	1.4	2.8	19.3	5.7
Femoral version	32	0.97	0.97–0.99	1.1	2.3	9.5	6.5
Femoral neck angle	32	0.96	0.93–0.99	1.1	2.2	136.7	5.5
Lateral center-edge angle	32	0.86	0.73–0.93	2.0	4.0	31.3	5.4
Alpha angle 3	31	0.78	0.59–0.89	2.6	5.2	41.5	5.5
Alpha angle 2	31	0.84	0.70–0.92	2.7	5.4	44.2	6.8
Alpha angle 1	31	0.86	0.72–0.93	3.1	6.1	49.2	8.2
Alpha angle 12	31	0.82	0.66–0.91	2.2	4.3	43.0	5.1

CI = confidence interval.

^a^For alpha angle, tester 1 and tester 2 that independently determined the scans from one hip was insufficient to measure due to bone marrow and soft tissue edema at the femoral head–neck junction, and therefore this scan was excluded from the analysis.

**Table IV.** Inter-rater reliability between novice and experienced testers

Variable	*n*^a^	Inter-tester		Inter-tester	
		Tester 1, Tester 3		Tester 2, Tester 3	
		ICC (2, 1)	95% CI	ICC (2, 1)	95% CI
Acetabular version	32	0.91	0.84–0.96	0.90	0.71–0.96
Femoral version	32	0.98	0.95–0.99	0.97	0.93–0.98
Femoral neck angle	32	0.93	0.86–0.97	0.93	0.86–0.96
Lateral center-edge angle	32	0.83	0.27–0.94	0.82	0.08–0.94
Alpha angle 3	31	0.54	0.22–0.75	0.63	0.35–0.80
Alpha angle 2	31	0.78	0.60–0.89	0.77	0.58–0.88
Alpha angle 1	31	0.52	0.21–0.74	0.50	0.17–0.72
Alpha angle 12	31	0.27	−0.20 to 0.74	0.23	−0.09 to 0.52

CI = confidence interval.

^a^For alpha angle, tester 1 and tester 2 that independently determined the scans from one hip was insufficient to measure due to bone marrow and soft tissue edema at the femoral head–neck junction, and therefore this scan was excluded from the analysis.

## DISCUSSION

The goal of this study was to develop a comprehensive, reliable and radiation-free method using MRI to quantify the femoral and acetabular morphology in subjects with IAHD and asymptomatic subjects. Using our methods, measurements such as AV, FV, FNA and the LCEA can be made reliably by both experienced and novice raters; however, the AA measurement was reliable only among experienced raters. These methods may be used in future studies to better capture the prevalence of bony abnormalities in symptomatic and asymptomatic populations and to better understand the natural history of IAHD and osteoarthritis. Additionally, these methods may facilitate surgical decision making and surgical planning when contemplating hip preservation surgery.

Unique to our methods is the standardization of pelvic orientation after image acquisition. Previous reports have established the need to standardize pelvic position to assess acetabular coverage of the femoral head [7–12]. In their study using CT, van Bosse *et al*. [11] concluded that pelvic obliquity >7° or pelvic tilt >4° would result in significant error in the measurement of AV. Dandachli *et al*. [10] reported similar findings reporting that for every 5° increase in pelvic anterior tilt, there was up to 5° decrease in acetabular anteversion. During method development, we determined that despite our efforts to standardized subject positioning prior to image acquisition, rotation of the pelvis was apparent in some of the original scans. We therefore developed methods to standardize pelvic orientation by correcting for pelvic coronal obliquity, axial rotation and sagittal tilt, thus limit the effect of pelvic positioning on our final measures. Correcting for these rotations should reduce error associated with malpositioning. We do not know how pelvic orientation after correction relates to the subject’s functional alignment or posture. This is a topic for future studies.

Our study is the first to demonstrate the reliability of raters with various levels of experience. Previous studies have assessed raters with extensive background and experience [22–26]. The results of our study suggest that some measurements can be performed reliably by novice raters with minimal clinical experience. We believe the methods we developed for training were useful in achieving high reliability in the novice rater, and may be useful when completing studies with large sample sizes, in which the assistance of novice raters may be required. When measuring AA, however, clinical expertise may be required as both intra-rater and inter-rater reliability were poor for the novice rater.

Among experienced raters, our findings were similar to previously published studies reporting the reliability of FV [24, 25, 27], LCEA [28] and AV [29] measurements using MRI. Ours is the first to report the reliability of FNA. The reported reliability using MRI to measure the AA varies. Similar to our experienced raters’ reliability which ranged from 0.78 to 0.86, Domayer *et al*. [26] and Sutter *et al*. [30] reported ICCs of 0.84 and 0.71, respectively; however, both used magnetic resonance arthrography (MRA) for the patients in their studies. We were able to achieve similar reliability without the addition of intra-articular contrast, which would not be recommended for use in asymptomatic controls. Nötzli *et al*. [22] assessed the AA using MRI and reported 7% variation in inter-rater agreement. In contrast, Lohan *et al*. [31], who used MRA, concluded that inter-rater agreement for AA measures was poor, stating up to 30% variation between the first and second measurements. Our study differed from the Lohan study in two ways. In our study, one rater performed the post-processing to obtain the radial images required to measure the AAs at various locations of the femoral neck. Although not explicitly stated, we assume in the study by Lohan that each rater selected the image slice to be measured. Selection of the image slice may increase the variability in their repeated measurements. Training also differed between studies. The raters in the study by Lohan ‘reviewed and discussed the approach of Nötzli et al’ prior to initiating the study. For our study, the two experienced raters along with an engineer developed a training manual providing specific decision-making rules. Prior to reliability testing, practice sessions were completed and the training manual revised to improve our performance. We believe the additional step of developing a written manual with specific instructions and use of practice sessions to discover areas of disagreement resulted in higher reliability.

The reliability of the LCEA and the AAs did not reach 0.90 level, a value recommended to ensure reasonable validity [21]. We reviewed the images after testing was completed and determined that for the LCEA, differentiating the acetabular labrum from the acetabular rim was challenging in a number of the cases. For our study, we did not provide additional images for cross referencing. Use of additional images in the same imaging plane would likely improve the ability to differentiate the labrum from the acetabular rim and thus improve the reliability of LCEA. For the AA, sizing of the circle on the femoral head likely contributed to the lower reliability. The femoral head is not completely spherical resulting in some disagreement in the appropriate size of the circle. To standardize circle selection, we used a Mose template along the sides of the femoral head that did not include the fovea. As a function of the software, the radius of the circle could not be increased or decreased by less than one pixel, therefore changes in the circle diameter would sometimes result in including too little or too much of the femoral head within the circle. A difference in the size of the circle between raters affects the exit point of the femoral neck from the circle, with a larger circle resulting in a smaller AA.

Our study findings should be considered in light of several limitations. To reduce error associated with image post-processing and to reduce burden on the raters, we chose to have one rater complete all post-processing. If these methods were to be used in multi-center studies, we would recommend that one rater at a central location complete the post-processing. Additional reliability testing would be needed if multiple raters were to be involved in the post-processing step. Completion of the measurement procedures is time consuming, a total of 50 min per subject to complete post-processing and measurement of bilateral limbs. The increased time to measure is offset by the short image acquisition time. Images from one hip, a 54-year-old female with IAHD, could not be used to assess the AA as both experienced raters determined that the images were not adequate to accurately make this measurement due to marrow edema at the femoral head–neck junction and adjacent soft tissues obscuring the cortical margin of the bone. This made it very difficult to determine the exact location the femoral neck exited the circle representing the femoral head. In this particular situation, a non–fat-suppressed 3D sequence would likely improve the ability to evaluate the cortical margin. We can only report on the reliability of these measures without the non–fat-suppressed 3D sequence. Six of our subjects had clinical signs and symptoms associated with IAHD; however, using only the images in our study, we are unable to determine if pathology exists. Additional sequences would be needed. The purpose of our study was to determine the bony morphology in painful and asymptomatic people, therefore the sequences were optimized for bony morphology only. Geometric distortion may occur with the use of MRI, which may affect the accuracy of our measures. The subjects of this study were either asymptomatic controls or people with IAHD who were not scheduled for surgical procedure or additional radiographic imaging. To avoid radiation exposure to our subjects, we chose not to collect radiographs or CT. We are, therefore, unable to assess the accuracy of our measures. The goal of this study was to develop reliable methods; therefore any distortion would have affected the raters’ measures equally. These methods may now be used in future studies to assess the accuracy of our methods.

In conclusion, MR provides a radiation-free measurement method that may be used to comprehensively assess femoral and acetabular morphology. We describe methods to standardize pelvic orientation after image acquisition to reduce error associated with subject malpositioning. Measurements such as AV, FV, FNA and the LCEA can be made reliably by both experienced and novice raters; however, the AA measurement was reliable only among experienced raters.
